# A case of severe intravascular haemolysis

**DOI:** 10.1002/jha2.520

**Published:** 2022-07-08

**Authors:** Alice Ching Ching Wong, Eudora Yu De Chow

**Affiliations:** ^1^ Department of Clinical Pathology Tuen Mun Hospital Hong Kong China; ^2^ Department of Pathology United Christian Hospital Hong Kong China

**Keywords:** *Clostridium perfringens*, liver abscess, spherocytosis

1

An 80‐year‐old man, who had a history of hypertension, diabetes, ischaemic heart disease and *Klebsiella* liver abscess with drainage performed 15 years ago, was admitted for right‐sided abdominal pain and symptomatic anaemia. Initial blood tests showed anaemia (88 g/L), leukocytosis (32.4 × 10 ^9^ /L), mildly deranged renal function tests and elevated total bilirubin to 178 µmol/L.

Peripheral blood smear (Figure 1) showed marked spherocytosis and moderate polychromasia, as well as neutrophilia with toxic neutrophils and occasional myelocytes.

**FIGURE 1:**
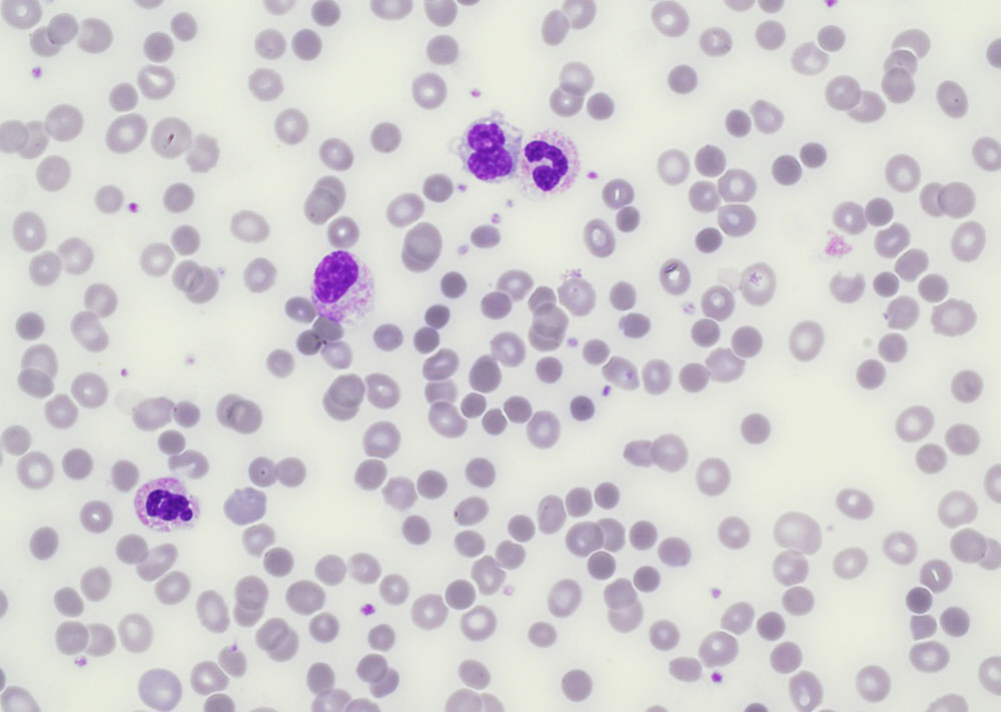
Peripheral blood smear showing spherocytosis with a myelocyte, neutrophils and a monocyte (60x magnification)

Subsequent blood tests performed, including clotting profile, liver and renal function tests, were reported to be unfit for analysis by automated analyzers due to gross haemolysis. The centrifuged plasma from the patient (left tube (Figure 2)) showed gross haemolysis and was reddish‐brown in colour, compared to the tube of centrifuged plasma obtained from a normal control on its right.

**FIGURE 2:**
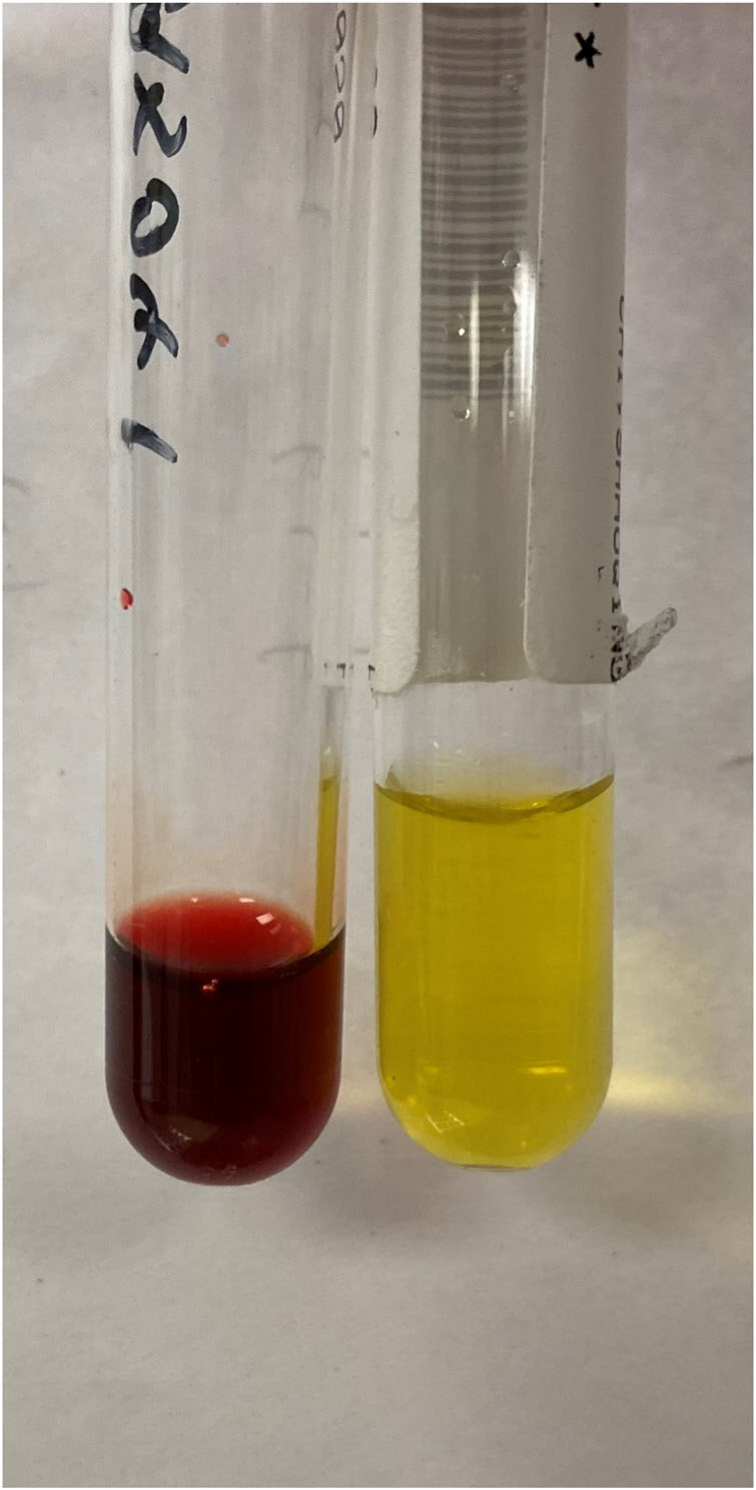
Grossly haemolyzed centrifuged plasma from the patient on the left, compared to centrifuged plasma from a normal control on the right

An urgent computed tomography scan of the abdomen showed a liver abscess with multiple gas locules (Figure 3) with perforation into the intraperitoneal cavity. His condition deteriorated rapidly due to septic shock with severe metabolic acidosis and succumbed within 8 hours of admission despite empirical antibiotics and active resuscitation.

**FIGURE 3:**
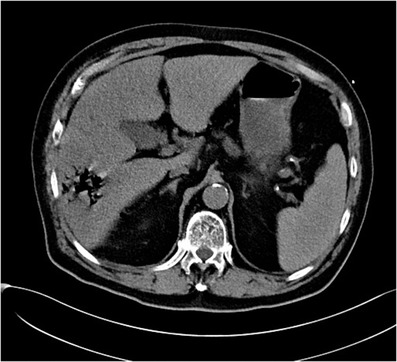
Computed tomography scan of the abdomen showing presence of a multiloculated liver abscess

Postmortem examination was performed. Blood culture grew *Escherichia coli (E. coli)*, while the pus from the liver abscess yielded heavy growth of *Clostridium perfringens (C. perfringens)*, moderate growth of *E. coli* and scanty growth of *Klebsiella*.

This was a case of severe intravascular haemolysis secondary to liver abscess due to *E. coli* and gas‐forming *C. perfringens*. Risk factors for *C. perfringens* septicaemia include diabetes, a recent history of gastrointestinal surgery and gastrointestinal malignancy [1]. It is important to have a high index of suspicion for *C. perfringens* septicaemia when coming across cases where blood samples are grossly haemolyzed with features of intravascular haemolysis including spherocytosis and polychromasia observed in the peripheral blood smear, as there can be rapid progression and high fatality (mortality up to ∼70%–100% [2]). Blood culture should be taken as soon as possible and sent for Gram stain, empirical antibiotics quickly administered, timely surgical intervention and aggressive supportive therapy should be provided for the best chance of cure.

## CONFLICT OF INTEREST

The authors declare that they have no conflict of interest.

## FUNDING

No funding has been received for this work.

## PERMISSION TO REPRODUCE MATERIAL FROM OTHER SOURCES

No reproduction of material from other sources has been utilized in the preparation of this manuscript.

## ETHICS STATEMENT

We comply to practice guidelines on research integrity and publishing ethics. No patient identifiable images or data have been included in the manuscript.

## Data Availability

Data sharing is not applicable to this article as no new data were created or analyzed in this manuscript.
